# Diabetes in Childhood

**Published:** 1890-01-11

**Authors:** 


### DIABETES IN CHILDHOOD.

Diabetes is a disease of rare occurrence in childhood, and in the opinion of most authorities, as Senatov, Schmitz, Budde, and Pavy, is almost invariably fatal sooner or later. Dr. Curt Stern has collected the records of 117 cases under fifteen years of age, six being infants of less than one year, and one having been diabetic from birth. Heredity played an important part, for even when the parents had not been subjects of glyconotics, other relatives, perhaps several, had been so. This was especially conspicuous in those instances in which two or more children of the same parents suffered, as in two reported by Dr. Pavy, where two such children of healthy parents could count two uncles, two aunts, and three great-aunts diabetic, and three of a family of five inherited the disease from mother and grandmother—that is, it was transmitted through three successive generations. Rossbach found that when there was not a clear history of diabetes there was always a neuropathic taint, as shown by epilepsy, insanity, gout, etc. Teschemacher frequently observed cerebral hæmorrhages *post mortem*, and Brieger has pointed out the occurrence of diabetes in connection with Werlhof's disease, malaria, typhus, and even measles. The symptoms in children are well marked, the wasting pallor and weakness are extreme, the skin dry, and prone to boils and eczematous eruptions, while the desire to micturate is incessant. The amount of urine varies with the age and the severity of the disease from 1,500 to 4,000, 5,000, 6,000, or even more ccms., while the little patients suffer from intense thirst and a craving for sweets or acids, and the appetite is excessive or perverted. The course of the disease, owing to the rapidity of the metabolic processes in childhood, is far shorter than in adults, death not seldom ensuing within a month, and existence being comparatively rarely prolonged beyond one year. If, however, one might judge from the published reports alone, it would seem not to be so fatal as has been supposed, for of the seventy-seven cases whose histories Dr. Stern was able to complete fourteen are stated to have recovered entirely, and in seven there was a permanent improvement. But recovery, though complete, may be only temporary, and so many relapses even after several years are recorded by Grantham, Heine, Schmitz, Frerichs, etc., that Senatov's assumption of an absolutely ultimate fatal termination, unless some other fatal disease intervene, seems justified, and Dr. Stern admits that could he have followed the subsequent history of the fourteen alleged recoveries he might have come to the same conclusion. As to the treatment, the conditions of growth in childhood preclude the strict dieting so successful with adults. Milk, plain or with cream added, or diluted with twice its volume of water, or thin gruel or barley water, or Carlsbad water, answer well. Older children may be dieted as adults under like circumstances. Every imaginable drug has been tried, but those only which have given any favourable results seem to be opium, salicylate of soda, and iron with the addition of some purgative.

